# Complex proximal femoral fractures in the elderly managed by reconstruction nailing – complications & outcomes: a retrospective analysis

**DOI:** 10.1186/1752-2897-1-7

**Published:** 2007-12-10

**Authors:** Ulfin Rethnam, James Cordell-Smith, Thirumoolanathan M Kumar, Amit Sinha

**Affiliations:** 1 Department of Orthopaedics, Glan Clwyd Hospital, Bodelwyddan, UK; 2 Department of Orthopaedics, Morriston hospital, Swansea, UK; 3 11 Ffordd Parc Castell, Bodelwyddan, Rhyl, LL18 5WD, UK

## Abstract

**Background:**

Unstable proximal femoral fractures and pathological lesions involving the trochanteric region in the elderly comprise an increasing workload for the trauma surgeon as the ageing population increases. This study aims to evaluate use of the Russell-Taylor reconstruction nail (RTRN) in this group with regard to mortality risk, complication rates and final outcome.

**Methods:**

Retrospective evaluation of 42 patients aged over 60 years who were treated by reconstruction nailing for proximal femoral fractures over a 4 year period.

**Results:**

Over two-thirds of patients were high anaesthetic risk (ASA > 3) with ischemic heart disease the most common co-morbidity. 4 patients died within 30 days of surgery and 4 patients required further surgery for implant related failure. Majority of patients failed to regain their pre-injury mobility status and fewer than half the patients returned to their original domestic residence.

**Conclusion:**

Favourable fixation of unstable complex femoral fractures in the elderly population can be achieved with the Russell-Taylor reconstruction nail. However, use of this device in this frail population was associated with a high implant complication and mortality rate that undoubtedly reflected the severity of the injury sustained, co-morbidity within the group and the stress of a major surgical procedure.

## Background

Locked intramedullary fixation has transformed the management of diaphyseal femoral fractures although the benefits compared to extramedullary devices in extracapsular hip fractures continue to be debated [1,2]. Complex proximal femoral fractures in the elderly population have become more prevalent as the ageing population increases. Such injuries typically include pertrochanteric hip fractures with extensive diaphyseal extension and subtrochanteric fractures, both of which present a considerable orthopaedic challenge due to co-morbidity and poor bone quality [3].

The Russell-Taylor Reconstruction Nail (RTRN) is a cannulated, stainless steel second generation cephalomedullary device. Its role extends beyond the simultaneous basicervical and diaphyseal injuries for which it was originally designed and successful use is reported [4-6]. The literature regarding its role in the elderly, however, who usually have low energy mechanisms and often dissimilar fracture configurations compared to the younger adult population, is more limited.

We report our experience of the Russell-Taylor reconstruction nail use in an exclusively elderly population with unstable inter-trochanteric and metastatic fractures involving the proximal femur. Our aim was to assess whether the reconstruction nail compared with the other intramedullary nails described in literature with regards to complications, mortality, re-operations and outcome. Could the reconstruction nail be considered a treatment option for unstable inter-trochanteric fractures in the elderly?

## Methods

Over a four year period (September 1999 to April 2003) 42 patients over 60 years of age with complex femoral fractures were treated by Russell-Taylor Reconstruction Nail fixation (RTRN).

Indications for the RTRN included unstable pertrochanteric fractures with diaphyseal extension, subtrochanteric fractures and pathological or impending fractures of the proximal femur. All patients treated using the Russell-Taylor Reconstruction Nail for proximal femur fractures during the study period were included. All procedures were performed at a busy district general hospital by Orthopaedic surgeons of differing experience and seniority. Data relating to patient demographics including co-morbidity, anaesthetic risk rating and injury mechanism were collected retrospectively (Table 1). Fractures were classified using the AO/ASIF system.

**Table 1:** Patient profile, co-morbidities, pre and post-op mobility status

**Case**	**Age**	**Sex**	**Mechanism of Injury**	**Type of injury**	**Co-morbidity**	**Pre-op mobility**	**Post-op mobility**
1	77	M	Fall	Low velocity	Chronic Obstructive Airway disease	Independent	Zimmer frame
2	88	F	Fall	Low velocity	Nil	1 stick	Zimmer frame
3	90	F	Fall	Low velocity	Supraventricular tachycardia	Independent	1 Stick
4	70	F	Fall	Low velocity	Nil	1 stick	Zimmer frame
5	89	F	Fall	Low velocity	Hypothyroidism	Independent	Zimmer frame
6	89	F	Spontaneous	Pathologic	Myocardial infarction/IHD	2 stick	Zimmer frame
7	77	M	Spontaneous	Pathologic	Lung Carcinoma	Zimmer frame	Independent
8	65	F	Fall	Low velocity	Ischaemic heart disease	Independent	Assistance
9	68	M	Fall	Low velocity	AF/COPD/Hypertension	Independent	Assistance
10	89	F	Fall	Low velocity	CCF/AF/Hypertension	Independent	Zimmer frame
11	77	M	Fall	Low velocity	IHD/PVD	1 stick	2 sticks
12	62	M	Fall	Pathologic	Metastatic prostate Carcinoma	Independent	Zimmer frame
13	64	M	Fall	Low velocity	Ischaemic heart disease	1 stick	Zimmer frame
14	78	F	Fall	Low velocity	Heart block, Pacemaker	1 stick	Wheelchair
15	83	F	Spontaneous	Pathologic	Metastatic breast Carcinoma	Independent	Zimmer frame
16	85	F	Fall	Low velocity	Nil	Independent	Wheelchair
17	67	M	Fall	Pathologic	Metastatic prostate Carcinoma	Independent	1 Stick
18	72	F	Spontaneous	Pathologic	Chronic renal failure	Independent	Zimmer frame
19	80	M	Fall	Low velocity	NIDDM/MI/Hypertension	Independent	Wheelchair
20	78	F	Fall	Pathologic	Metastatic breast Carcinoma	1 stick	Wheelchair
21	91	M	Fall	Low velocity	IHD/CCF/PE	Independent	N/A
22	79	M	Fall	Low velocity	Paget's disease/IHD/Hypertension	1 stick	1 Stick
23	75	F	Fall	Low velocity	Hypertension	Independent	Independent
24	69	M	Fall	Pathologic	Metastatic prostate Carcinoma	Independent	N/A
25	75	F	Fall	Low velocity	IHD/AF/PVD	Independent	Independent
26	70	F	Impending	Pathologic	Metastatic breast Carcinoma	Independent	Zimmer frame
27	81	F	Fall	Low velocity	Hypothyroidism	Independent	Independent
28	69	M	Fall	Low velocity	Hypertension/AAA repair	Independent	Zimmer frame
29	88	M	Fall	Low velocity	IHD/Hypertension	1 stick	Zimmer frame
30	81	F	Fall	Low velocity	AF/NIDDM/Stroke	Independent	1 Stick
31	72	F	Spontaneous	Pathologic	Lung Carcinoma	Independent	Zimmer frame
32	81	F	Fall	Low velocity	Hypertension	Zimmer frame	2 sticks
33	68	F	Fall	Low velocity	Chronic Obstructive Airway disease	Independent	N/A
34	90	F	Fall	Low velocity	Hypertension/IHD	Zimmer frame	N/A
35	80	F	Impending	Pathologic	Metastatic breast Carcinoma	Independent	Zimmer frame
36	90	F	Fall	Low velocity	Hypertension	Zimmer frame	Independent
37	77	F	Fall	Low velocity	Nil	Independent	Zimmer frame
38	86	M	Spontaneous	Pathologic	Multiple myeloma	1 stick	Zimmer frame
39	94	F	Fall	Low velocity	Hypertension	1 stick	Zimmer frame
40	72	M	Fall	Low velocity	Paget's disease	Independent	Zimmer frame
41	89	F	Fall	Low velocity	IHD/CCF/MR	Independent	Zimmer frame
42	68	F	Spontaneous	Pathologic	Metastatic breast Carcinoma	Wheelchair	Wheelchair

Most fractures were treated by closed reduction methods using a traction table under fluoroscopic guidance. However, open techniques and cerclage wiring was performed for selected fracture types that were irreducible using standard closed techniques. Patients were routinely mobilized full weight bearing as tolerated in the post-operative period. Operative duration, peri-operative and postoperative complications were assessed (Table 2). Pre-operative mobility was assessed on admission from a thorough history and compared to the post-operative mobility gained (Table 1).

**Table 2:** Complications and post-operative mortality

**Patients**	**Surgical time (min)**	**Intra-op Complications**	**Post-op complications**	**Mortality <6 months**
1	65	Nil	Nil	Alive
2	113	Nil	Nil	Alive
3	103	Nil	Nil	Alive
4	140	Nil	Excision of prominent fragment	Alive
5	89	Nil	Nil	Alive
6	85	Nil	Nil	Alive
7	130	Fracture medial cortex femur	Nil	Died 2 weeks post-op
8	167	Nil	Nil	Alive
9	255	Difficult access to piriformis	Nil	Alive
10	91	Nil	Nil	Alive
11	155	Bleeding	Nil	Alive
12	244	Distal locking not possible	Deep vein thrombosis	Alive
13	92	Difficult access to piriformis	Wound infection	Alive
14	160	Nil	Nil	Alive
15	113	Nil	Nil	Died 10 weeks post-op
16	141	Open reduction	Nil	Alive
17	89	Nil	Nil	Died 8 weeks post-op
18	90	1 proximal screw	Nil	Alive
19	140	Cerclage for comminution	Nil	Alive
20	189	Nil	Post-op ileus	Alive
21	86	Nil	Distal screw backout	Alive
22	126	Difficult access to piriformis	Renal failure, death	Died 10 days post-op
23	185	Open reduction	Nil	Alive
24	182	Nil	Nil	Alive
25	96	MI	Death 2 hours post-op	Died 2 hours post-op
26	104	Nil	Deep vein thrombosis	Alive
27	129	Nil	Proximal screw backout, wound infection	Alive
28	170	Open reduction	Non-union, implant frature	Alive
29	135	Nil	Nil	Alive
30	141	Varus reduction	Fracture displacement	Alive
31	119	Nil	Nil	Alive
32	145	Nil	Nil	Alive
33	98	Nil	Nil	Alive
34	165	Nil	Post-op LVF & death	Died 1 day post-op
35	140	Nil	Post-op death	Died 1 week post-op
36	132	Nil	Excision of prominent fragment	Died 3 months post-op
37	114	Nil	Proximal screw backout	Alive
38	88	Nil	Unicortical fracture around nail	Alive
39	160	Nil	Wound infection	Alive
40	143	Varus reduction	Nil	Alive
41	130	Open reduction	Proximal screw migration	Alive
42	91	Nil	Nil	Alive

## Results

42 patients over 60 years of age (mean: 78 years, range 62 – 94 years) with complex femoral fractures treated by Russell-Taylor Reconstruction Nail were included. There were 27 female and 15 male patients in the cohort. 29 fractures were a consequence of low energy falls and 13 were pathological (31%). The commonest pathological fracture was due to metastatic breast carcinoma (Table 3). Spiral subtrochanteric fractures classified as AO/ASIF 32-A1.1 was the most common fracture configuration although this comprised 38% of all types (Table 4).

**Table 3:** Incidence of pathological fractures in the study

Metastatic breast carcinoma	5
Metastatic prostatic carcinoma	3
Metastatic bronchogenic carcinoma	2
Multiple myeloma	1
Paget's disease	2

**Table 4:** Fracture type (AO/ASIF Classification)

Type of fracture	AO/ASIF Category	Number of patients
**Pertrochanteric multifragmentary (>1 cm below lesser trochanter)**	31-A2.3	3
**Intertrochanteric multifragmentary**	31-A3.3	2
**Simple spiral subtrochanteric**	32-A1.1	16
**Simple oblique subtrochanteric**	32-A2.1	7
**Simple transverse subtrochanteric**	32-A3.1	6
**Wedge, spiral subtrochanteric**	32-B1.1	3
**Wedge, bending subtrochanteric**	32-B2.2	1
**Wedge, fragmented subtrochanteric**	32-B3.3	2
**Impending pathological fracture**	N/A	2

Anaesthetic risk, as graded by the American Society of anaesthesiologists, was high (median ASA grade 3 in 57%) as the majority of patients had co-morbidities. Ischaemic heart disease was the most common associated medical condition.

The mean operative duration was 131.6 ± 41.1 minutes (range: 85–255 minutes, 95% confidence interval 119 – 144.2 minutes), which reflected surgical experience, problems associated with fracture reduction and intra-operative technical difficulties most commonly relating to piriform fossa access and locking (Table 2). In 13/42 (31%) patients intra-operative difficulties were encountered (Table 2).

4 of 42 patients (9.5%) died within thirty days of surgery, 2 from peri-operative cardiac events, 1 from renal impairment and another from diverticular peritonitis. Of the patients who died, 2 patients were from the low energy fall group while 2 patients had metastatic pathological fractures.

Post-operative complications were encountered in 18/42 patients (42.8%). 3 patients developed wound infection one was a superficial wound infection that settled with antibiotics while the other 2 patients required surgical debridement.

**Figure 1:**
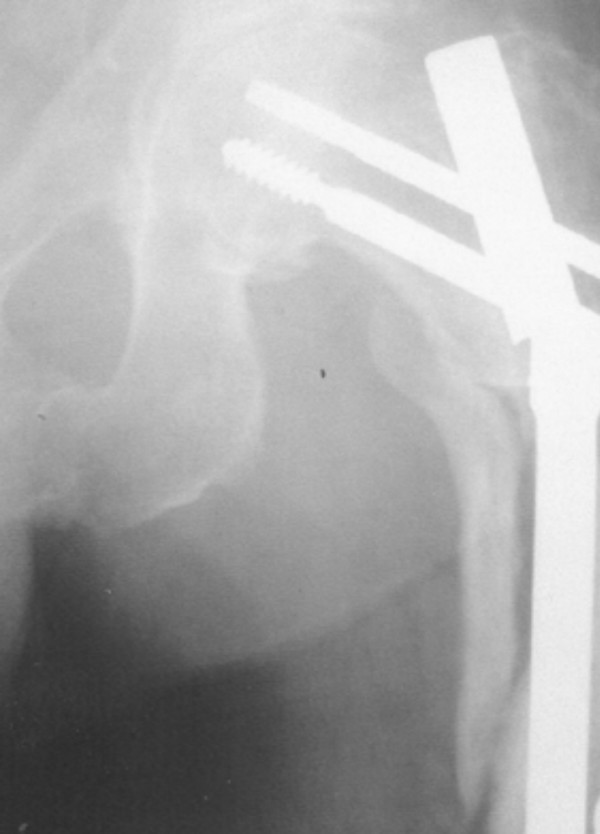
Implant failure at 13 months post-op.

**Figure 2:**
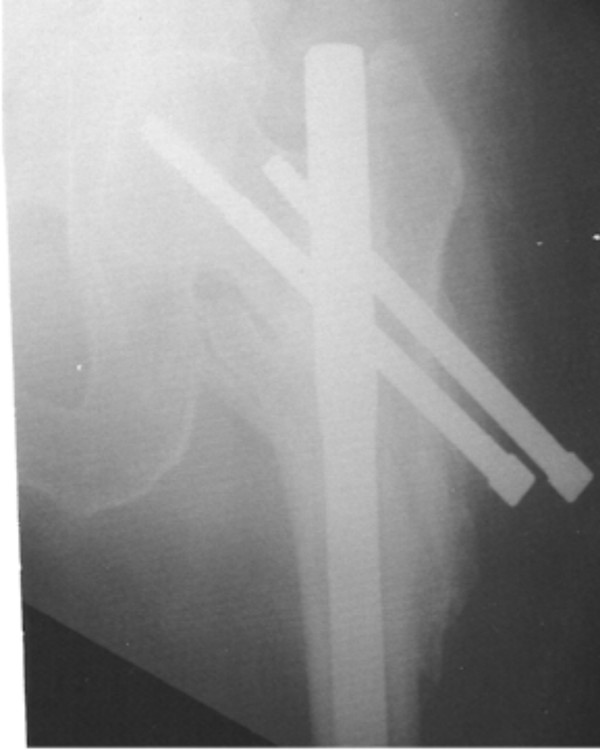
Reversed "Z" phenomenon ("Back out" of screws causing impingement symptoms).

**Figure 3:**
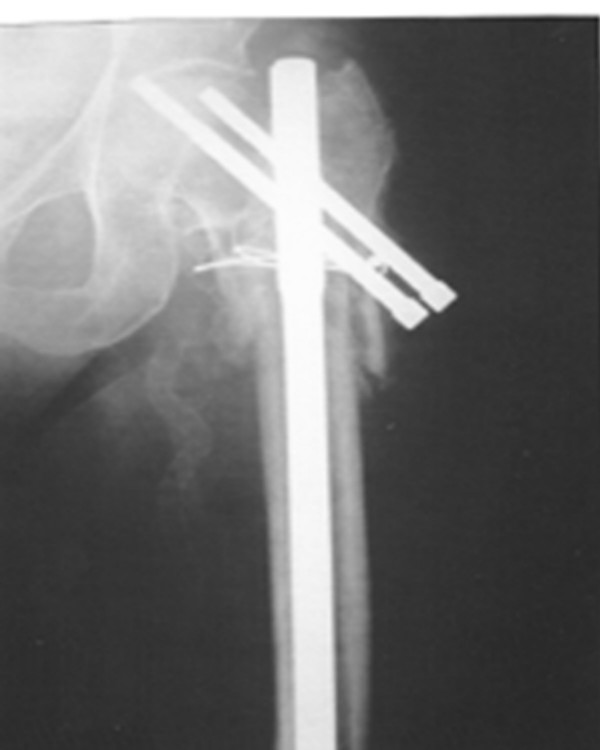
"Z" phenomenon. (Proximal migration of screw into hip joint).

Additional surgery was necessary in 7 patients (16.6%). One patient had implant failure at 13 months due to non-union (Figure 1) which was treated by exchange reconstruction nailing and the fracture united uneventfully subsequently. 3 patients required proximal locking screw removal, 2 for "backout" causing impingement symptoms (Reversed "Z" effect) (Figure 2), and 1 for proximal migration into the hip joint ("Z" effect) which was identified on serial radiographs and removed before intra-pelvic or abdominal injury occurred (Figure 3). 2 patients needed surgery for excision of prominent bone fragment. (Table 2)

71% of patients (30/42) had lived independently at home prior to their injury whereas only 31% (13/42) returned to their former domestic residence at discharge. Likewise, 26/42 (62%) patients had been independently ambulant but only 5 (12%) managed to achieve mobility without walking aids after surgery.

8/42 patients (19%) died within 6 months of the surgery. The fracture union time was 14.8 ± 3.76 weeks (Range: 8 – 24 weeks, 95% Confidence interval: 13 – 16 weeks).

## Discussion

Non operative management of pertrochanteric fractures was practised prior to introduction of fixation devices. In the elderly patient this approach was fraught with high complication and mortality rates [7]. Operative treatment of these fractures in the early allowed early rehabilitation and the best chance for functional recovery.

The implants for fixation of pertrochanteric fractures have evolved from fixed angle nail plate devices to the widely used to the newer generation cephalomedullary nails. The sliding hip screw is a tried and tested device for fixation of these fractures with excellent results reported [7]. In unstable and reverse oblique inter-trochanteric fractures, the intramedullary devices have an advantage of being load sharing with smaller bending moments as their position is closer to the mechanical axis of the femur as compared to the sliding hip screw. Intramedullary devices have a shorter lever arm and have reduced tensile strain on the implant reducing the risk of implant failure.

Various intramedullary devices have been used for fixation of these fractures – Ender's nail, the Russel Taylor reconstruction nail, the Gamma nail, proximal femoral nail and the AMBI nail. Studies comparing the gamma nail and sliding hip screw have found higher incidence of complications and re-operation rates with the gamma nail and no difference in long term functional outcomes [8]. Most peri-operative complications while using the Gamma nail were related to poor technique. The advantages with the Gamma nail were early mobilisation and full weight bearing [9]. The surgical technique with the Russel Taylor reconstruction nails has been known to be demanding with high post-operative complications [6]. Studies were the Proximal Femoral Nail (PFN) were used cited high intra-operative and post-operative complications. The PFN was also associated with high re-operation rates [10,11]. The intramedullary nails are better implants for unstable reverse oblique fractures while the sliding hip screw better for stable inter-trochanteric fractures [1]. No difference between the Gamma nail and the PFN were seen in terms of fracture healing, re-operation and mortality rates [12]. Shorter operating times, fewer blood transfusion and shorter hospital stay have been found while using intramedullary nails as compared to the 95 fixed angle screw plate for unstable intertroachanterics fractures. Intramedullary nails have been advocated for reverse oblique fracture of the inter-trochanteric region in the elderly [13]. A prospective randomised trail comparing different intramedullary nails for treatment of pertrochanteric fractures concluded that the AMBI nail was the gold standard while the PFN had the most complications and longest operation times [14]. The general consensus in the literature is that the sliding hip screw is superior for fixation of stable inter-trochanteric fractures while the intramedullary nails are best reserved for the unstable and reverse oblique variety.

The patient cohort studied in our study demonstrated features typical of their demographic group including high levels of concomitant medical disease, a female predominance and low energy injury mechanisms i.e. simple falls. This group differs markedly from the younger adult population who generally sustain higher energy trauma and multiple injuries for which the conventional management for complex proximal femoral fracture is intramedullary fixation. The frailty of the elderly undoubtedly predisposes this group to high perioperative mortality rate due to poorer physiological reserve.

The Russell-Taylor reconstruction nail provided satisfactory fixation in the majority of elderly patients with complex and unstable proximal femoral injuries. This implant provided the opportunity for early mobilisation although most patients did not return to their pre-injury level of independence or mobility. The reconstruction nail used had the biomechanical benefits of intramedullary fixation compared to extramedullary techniques [2]. However, implant-related failures did occur and revision surgery was required at levels consistent with other studies [4-6]. Actual mechanical failure of the nail occurred in only one patient who developed a non-union leading to implant failure.

A more common event was migration of the oblique proximal interlocking screw. This may arise due to the poor bone density of the femoral head which limited screw purchase and reflects one of the many problems associated with fixation in elderly, osteoporotic bone [3]. Migration of the interlocking screws occurs within the nail as these do not secure rigidly within the device itself and is described in the literature as "Z" effect (Proximal migration of the proximal screw) and the "Reversed Z" effect (Distal migration of the proximal screw) [11,15].

We found use of this implant to be technically challenging resulting in highly variable and long operating times particularly for the less experienced surgeons. Although this places high physiological demands on frail, elderly patients with co-morbidity who are already at high mortality risk from their injury [16] the reconstruction nail aided early rehabilitation of function and reduced the morbidity associated with prolonged immobilization. The intra-operative and post-operative complications, re-operation and mortality rates in our study were lesser than that were encountered in studies were other nails (Gamma nail, PFN, Trochanteric Gamma nails) were used.

Surgical management of proximal femur fractures in the elderly is a challenging prospect as there is no ideal fixation method. All fixation methods available are fraught with complications, increased morbidity and mortality. The reconstruction nail could be used as an intramedullary fixation device for these fractures despite the high morbidity, complications and mortality encountered in our study.

## Conclusion

The locked reconstruction femoral nail permitted adequate fixation of unstable proximal femoral injuries in the elderly group studied. This procedure was associated with inherent mortality and complication risks which could be related to the bone quality and co-morbidity in the elderly. We feel that the reconstruction nail compares well with the newer intramedullary nails for the treatment of proximal femur fractures in the elderly.

## Competing interests

The author(s) declare that they have no competing interests.

## Authors' contributions

UR was involved in collecting patient details, reviewing the literature, drafted and proof read the manuscript. JCS was involved in collecting patient details, reviewing the literature, drafted and proof read the manuscript. TMK was involved in data collection and proof reading the manuscript. AS is the senior author and was responsible for final proof reading of the article. All authors have read and approved the final manuscript.
